# A Case of Rupture of the Uterus with the Escape of the Fœtus into the Abdomen

**Published:** 1848-02

**Authors:** James M. Larkins

**Affiliations:** Charlotte, Tennessee


ART. II.—*A Case of Rupture of the Uterus with the escape of the Fœtus into the Abdomen.* By JAMES M. LARKINS, M.D., of Charlotte, Tennessee.

Dr. Hudson, a physician of highly respectable standing in this county (Dickson), was called on the 7th of August last to the house of Mr. N. to see a servant woman. This woman had been in a lingering condition for sometime previous to the doctor's visit, but had suddenly got worse. Neither her master nor any of the family could give a satisfactory account of her condition, and that given by the patient was false, for she denied being in the family way, notwithstanding her husband was living, and she the mother of several children. Her account of the case was this: she said her health had been about as usual until some two or three days before the doctor's visit, when she commenced swelling up so that the abdomen was greatly enlarged; pain in the loins, and frequent calls to make water, but a few drops being passed at any time.

The doctor supposed the case to be one of pregnancy, but on receiving the above information, and from the group of symptoms present, mistook the case for dysuria. An effort was made to introduce a catheter, but as no water passed he concluded that he had failed in the introduction of the instrument. A second physician was now called in, who concurred in the nature of the case; other efforts were accordingly made at the introduction of the catheter but failed in bringing away any urine. The failure now they supposed must have resulted from their having used the gum elastic catheter. I was sent for as having a silver instrument. Frequent attempts were made by Dr. Hudson and myself to draw off the urine, only few drops following each effort. As the patient was evidently sinking we determined to puncture the bladder through the parietes of the abdomen. Previously however to resorting to this last method, we fully satisfied ourselves of the fact that if the patient was pregnant the fœtus was dead. The operation was followed by a discharge of a most abominably offensive matter, a combination of pus and liquor amnii. That night the patient died. Next day the body was examined by Dr. Hudson and myself, when the obscurity of the case was explained. The uterus was ruptured; the head of the fœtus having escaped into the abdomen. From the appearance, the rupture of the uterus had taken place about the seventh month of gestation. The rupture was in the left angle, between the left Fallopian tube and the fundus; the margins of the rupture were closely constricted around the neck of the fœtus; the head of the child was collapsed, and in a half putrid condition. The parietes of the uterus were very much thickened; the margins of the rupture were fungous; an abscess containing two or three ounces of pus was situated in the right iliac region; every convolution of the bowels was filled with pus of a cream color. The bladder was in a healthy condition, and not distended with urine. The uterus, together with the abscess above mentioned, pressed upon it so that it could not retain the urine when this fluid passed into it. We were not able to satisfy ourselves at the time what was the cause of the rupture, but I have since learned that the patient had taken something to prevent conception. Whether by this procedure she did any injury to the uterus is not known; my opinion of the case is, that the uterus had an ulcer situated in that part which afterwards became ruptured, and as this organ enlarged to accommodate itself to the growth of the fœtus, that part of the parietes being unable from disease to undergo greater distention, a rupture took place. No part except the head escaped through the rent; the body, placenta, and liquor amnii were all retained, and the child died, probably, from the constriction of the walls of the uterus around its neck.

Charlotte, August 8th, 1847.

